# Mushroom body miscellanea: transgenic *Drosophila* strains expressing anatomical and physiological sensor proteins in Kenyon cells

**DOI:** 10.3389/fncir.2013.00147

**Published:** 2013-09-23

**Authors:** Ulrike Pech, Shubham Dipt, Jonas Barth, Priyanka Singh, Mandy Jauch, Andreas S. Thum, André Fiala, Thomas Riemensperger

**Affiliations:** ^1^Department of Molecular Neurobiology of Behavior, Georg-August-Universität GöttingenGöttingen, Germany; ^2^Department of Biology, Universität KonstanzKonstanz, Germany

**Keywords:** *Drosophila melanogaster*, mushroom body, Kenyon cells, optical calcium imaging, GRASP, photoactivation, transgene expression, neuroanatomy

## Abstract

The fruit fly *Drosophila melanogaster* represents a key model organism for analyzing how neuronal circuits regulate behavior. The mushroom body in the central brain is a particularly prominent brain region that has been intensely studied in several insect species and been implicated in a variety of behaviors, e.g., associative learning, locomotor activity, and sleep. *Drosophila melanogaster* offers the advantage that transgenes can be easily expressed in neuronal subpopulations, e.g., in intrinsic mushroom body neurons (Kenyon cells). A number of transgenes has been described and engineered to visualize the anatomy of neurons, to monitor physiological parameters of neuronal activity, and to manipulate neuronal function artificially. To target the expression of these transgenes selectively to specific neurons several sophisticated bi- or even multipartite transcription systems have been invented. However, the number of transgenes that can be combined in the genome of an individual fly is limited in practice. To facilitate the analysis of the mushroom body we provide a compilation of transgenic fruit flies that express transgenes under direct control of the Kenyon-cell specific promoter, mb247. The transgenes expressed are fluorescence reporters to analyze neuroanatomical aspects of the mushroom body, proteins to restrict ectopic gene expression to mushroom bodies, or fluorescent sensors to monitor physiological parameters of neuronal activity of Kenyon cells. Some of the transgenic animals compiled here have been published already, whereas others are novel and characterized here for the first time. Overall, the collection of transgenic flies expressing sensor and reporter genes in Kenyon cells facilitates combinations with binary transcription systems and might, ultimately, advance the physiological analysis of mushroom body function.

## Introduction

The mushroom body of the arthropod brain is a prominent brain structure that has attracted the attention of neuroscientists for more than 160 years (Dujardin, 1850; Kenyon, 1896; Strausfeld et al., 1998; Fahrbach, 2006; Strausfeld et al., 2009; Pech et al., 2013). Functionally, the mushroom body has been implicated in a variety of adaptive behaviors, e.g., associative olfactory learning (Davis, 1993; Heisenberg, 2003; Fiala, 2007), locomotor activity (Martin et al., 1998), or sleep (Bushey and Cirelli, 2011). The information-processing properties of mushroom bodies remain, however, unclear and are the subject of much current research. The mushroom body of the fruit fly *Drosophila melanogaster* consists of ~ 2000–2500 intrinsic neurons per hemisphere called Kenyon cells (Technau, 1984; Aso et al., 2009). Kenyon cells extend their dendrites at the calyx, the main sensory input region of the mushroom body. Olfactory projection neurons originating from the antennal lobes, the primary olfactory neuropils of the insect brain, transmit odor information to the ipsilateral mushroom body calyx, where they form large presynaptic boutons (Yasuyama et al., 2002; Leiss et al., 2009; Butcher et al., 2012). Kenyon cell dendrites contact these boutons and integrate odor information from many olfactory projection neurons (Caron et al., 2013). The Kenyon cells extend long axons into the protocerebrum, and the parallel bundles of axons together form the peduncle and the lobes of the mushroom body, the latter being both pre- and postsynaptic to mushroom body extrinsic neurons that provide afferent input to and/or efferent output from Kenyon cells (Ito et al., 1998; Tanaka et al., 2008). The mushroom body consists, however, not of an entirely homogeneous population of Kenyon cells, but rather one that can be subdivided into different subtypes according to different axonal projections (Yang et al., 1995; Crittenden et al., 1998; Aso et al., 2009). The α/β-lobe and α′/β′-lobe Kenyon cells bifurcate and extend one collateral into the dorsal-anterior direction and one toward the midline of the brain. The parallel, bundled axons collectively form the vertical α/α′-lobes and the horizontal β/β′-lobes. A third group of Kenyon cells does not divide their axons and form the γ-lobes that are positioned anterior to the β/β′-lobes. These Kenyon cell subgroups can be further subdivided into those forming core and surface, posterior and anterior regions of the mushroom body (Tanaka et al., 2008; Aso et al., 2009).

Diverse aspects of the function of mushroom bodies have been investigated in a variety of insect species. Physiological properties of individual Kenyon cells and mushroom body extrinsic neurons have been analyzed in larger insects that are amenable to intracellular electrophysiological recordings, e.g., in cockroaches (Li and Strausfeld, 1997; Mizunami et al., 1998; Li and Strausfeld, 1999; Demmer and Kloppenburg, 2009) or locusts (Stopfer et al., 2003; Cassenaer and Laurent, 2007). Honey bees represent excellent model organisms to study behavioral complexity and behavioral, experience-dependent plasticity in insects. In this context, the mushroom body and associated neurons have been investigated in detail, e.g., using pharmacological approaches (Hammer and Menzel, 1998; Louis et al., 2012), local anesthetics (Devaud et al., 2007), local cooling (Erber et al., 1980), optical Ca^2+^ imaging (Faber and Menzel, 2001; Szyszka et al., 2005), or electrophysiological recordings (Hammer, 1993; Strube-Bloss et al., 2011; Hussaini and Menzel, 2013).

In *Drosophila*, experimental approaches that involve physical intrusions, e.g., local injections, or the precise insertions of electrodes, are difficult due to the small size and fragility of the brain and its neurons. However, *Drosophila melanogaster* has been developed into an animal model system distinguishable from other insects by the feasibility to express transgenes in dedicated subpopulations of neurons (Olsen and Wilson, 2008; Venken et al., 2011). Transgenes that can help to analyze neuronal structure and/or function are, first, anatomical markers, e.g., cytosolic or subcellular anchored fluorescence proteins. Second, reporter proteins can be expressed to monitor physiological parameters of neuronal function, e.g., intracellular Ca^2+^ dynamics (Fiala et al., 2002; Riemensperger et al., 2012) or second-messenger signaling (Lissandron et al., 2007; Shafer et al., 2008). Third, effector proteins can be expressed to manipulate specific aspects of neuronal functioning. Membrane potentials can be affected through the expression of ion channels that are either constitutively in an open state (Nitabach et al., 2002), or dependent on external factors like temperature (Hamada et al., 2008) or light (Schroll et al., 2006). Likewise, chemical synaptic transmission can be prevented either constitutively (Sweeney et al., 1995; Baines et al., 2001) or reversibly (Kitamoto, 2001).

To target the expression of transgenes to specific neuronal populations, several bipartite expression systems have been invented for *Drosophila*, e.g., the Gal4-UAS-system (Brand and Perrimon, 1993), the lexA/lexAop-system (Lai and Lee, 2006), and the Q-system (Potter and Luo, 2011). These binary transcription systems typically divide into two transgenic fly strains—one for the desired transgene be expressed and the other for spatio-temporal control of the transgene. Large collections of “driver lines,” e.g., Gal4 strains or lexA strains, have been assembled and made available so that a variety of neurons can be targeted, in some cases rather selectively (e.g., Jenett et al., 2012). Sophisticated additional genetic techniques have even upgraded the possibility of restricting transgene expression in space and time, e.g., with the help of heat-inducible promoters, the additional expression of repressors of gene expression, or through expression of recombinases (Duffy, 2002; Pfeiffer et al., 2008, 2010; Venken and Bellen, 2012). These multipartite expression strategies have helped to refine the expression of transgenes to very few neurons of interest. In addition, several binary transcription systems can be combined to express different transgenes in different neuronal subpopulations, e.g., to monitor the activity of a certain neuronal population while manipulating a different subset of neurons. Of course, the number of transgenes that can be simultaneously expressed in one individual fly is limited. To enhance the versatility of transgene expression in order to analyze the anatomy and/or function of the mushroom body we have created a number of flies that express transgenes under direct control of the promoter mb247 (Schulz et al., 1996; Zars et al., 2000). Two copies of the mb247 promoter drive gene expression in all types of Kenyon cells (Riemensperger et al., 2005; Pech et al., 2013) with relatively high specificity. Fluorescent markers, physiological sensor proteins, and effector proteins are expressed under control of the mb247 promoter. Binary transcription systems are, therefore, still available to express additional transgenes in complementary neuronal populations. The particular transgenes expressed are suitable to be combined with each other, thereby enabling analyzing distinct anatomical and functional neuronal parameters simultaneously.

## Materials and methods

### *Drosophila* stocks

Flies were raised on standard cornmeal-agar food at 25°C, 60% relative humidity and a 12 h light-dark cycle. The following published *Drosophila* strains were used: mb247-DsRed (Riemensperger et al., 2005), mb247-DsRed; mb247-splitGFP11, UAS-splitGFP1-10 (Pech et al., 2013), c305a-Gal4 (Krashes et al., 2007), TH-Gal4 (Friggi-Grelin et al., 2003), mb247-Gal4 (Zars et al., 2000), mb247-LexA::VP16 (Pitman et al., 2011), UAS-mcd8-GFP (Lee and Luo, 1999). UAS-FRT-Stop-FRT-mcd8-GFP (Yu et al., 2010), LexAop-GFP (Tamura et al., 2010), and actin-FRT-Stop-FRT-Gal4; UAS-GFP; (Pignoni and Zipursky, 1997).

### Generation of novel drosophila strains

To generate mb247-C3paGFP flies the C3paGFP DNA was amplified from the genomic DNA of UAS-C3paGFP flies (Ruta et al., 2010) by linker PCR using the primers ATCAGATCTCAAAAATGGTGAGCAAGGGCGAGGA and AAGAAATGCGGCCGCTTACTTGTACAGCTCGTCC, producing BglII and NotI restriction sites. For generating flies expressing the fluorescence Ca^2+^ indicators G-GECO1.1, G-GECO1.2, and R-GECO1.0 (Zhao et al., 2011) under control of the mb247 promoter, the DNA sequences from the original pCMV vectors (addgene # 32444, 32445, 32446) were amplified by linker PCR using the primers ATCAGATCTCAAAAATGGTCGACTCTTCACGTCG and AAGAAATGCGGCCGCCTACTTCGCTGTCATCATTT producing BglII and NotI restriction sites. To generate the mb247-flippase fly strain the flippase-IRES-flippase sequence was amplified, using linker PCR from the original vector (Bohm et al., 2010), using the primers GAAGATCTTCCACCATGCCACAATTTGGTATATTATG and GAAGGCCTTCTTATATGCGTCTATTTATGTAGG, producing BglII and StuI restriction sites, respectively. To drive expression of GCaMP3.0 (Tian et al., 2009) under control of the mb247 promoter, the 6xHis-tagged GCaMP3.0 DNA was amplified by linker PCR from the purified genomic DNA obtained from UAS-GCaMP3.0 flies (Tian et al., 2009) using the primers ATCAGATCTCAAAAATGGGTTCTCATCATCATCATCATCATG and ATCGCGGCCGCTTACTTCGCTGTCATCATTTGTACAAACTCTTC, producing BglII and NotI restriction sites. For generating mb247-Synapto-pHluorin flies the Synapto-pHluorin DNA was amplified by linker PCR from the original vector (Miesenböck et al., 1998) using the primers GAAGATCTACGCGTGCCACCATGTCG and ATTTGCGGCCGCCTAGATTAACCGGTTTT, producing BglII and NotI restriction sites. All DNA constructs were inserted into the pCaSpeR vector containing two copies of the mb247 promoter fragment that was originally obtained from Martin Heisenberg's laboratory and fully sequenced in the course of this study. Germ-line transformation was performed by the BestGene company (Chino Hills, CA).

### Immunohistochemistry

Adult brains were dissected in ice-cold Ringer's solution containing 5 mM Hepes, 130 mM NaCl, 5 mM KCl, 2 mM MgCl_2_, 2 mM CaCl_2_, pH = 7.3 (Estes et al., 1996), fixed for 2 h on ice in 4% paraformaldehyde dissolved in phosphate buffered saline (PBS), and washed three times in PBS containing 0.5% Triton X-100 (PBST) for 20 min each. After overnight pre-incubation in PBST containing 2% bovine serum albumin (blocking solution) at 4°C, brains were incubated for 5 h at room temperature with the primary antibodies diluted in blocking solution. For experiments with splitGFP the brains were pre-incubated in blocking solution containing 0.1% bovine serum albumin and 5% normal goat serum for 2 h at 4°C. The following antibodies were used: mouse anti-nc82 against Bruchpilot (provided by Erich Buchner) diluted 1:5, rat anti-RFP (5F8, Chromotec) diluted 1:300, and rabbit anti-GFP (A6455, Invitrogen) diluted 1:200. Subsequently, brains were washed three times for 20 min each in PBST and incubated overnight at 4°C with the secondary antibodies: goat anti-mouse conjugated with Cy3 (A1101, Invitrogen), goat anti-rabbit conjugated with Alexa Fluor 488 (A11034, Invitrogen) and goat anti-rat conjugated with Cy3 (A10522, Invitrogen), all diluted 1:300. To visualize reconstituted split-GFP, brains were incubated with anti-GFP-20 (Sigma, G6539) diluted 1:200 in blocking solution at 4°C overnight and, after three washing steps at room temperature in PBST, with anti-mouse conjugated to Alexa488 diluted 1:250 in blocking solution at 4°C overnight. Afterwards, brains were washed three times in PBST for 20 min each, washed in PBS, overnight at 4°C, embedded in Vectashield (Vector Laboratories) and images were acquired using a confocal laser scan microscope (SP2, Leica) equipped with an Apochromat 20 × water immersion objective (NA = 0.7). Images were analyzed using ImageJ.

### Photoactivation of photoactivatable GFP

For photoactivating photoactivatable GFP (paGFP) and the visualization of resulting *in vivo* fluorescence patterns, the brains of 7-day-old female flies were dissected and scanned using a Zeiss LSM7 MP two-photon microscope equipped with a Zeiss w-plan Apochromat 20 × water immersion objective (NA = 1.0), at an excitation wavelength of 950 nm, a pixel dwell of 2.3 μs and a line average of 4. PaGFP and red DsRed fluorescence were recorded simultaneously using a dichroic mirror in combination with 500–550 and 575–610 nm emission filters. To photoactivate paGFP, a small region within one of the two mushroom bodies was chosen, indicated by the mb247-DsRed fluorescence as a landmark, and subsequently this region was scanned at 760 nm with a laser power of 5% and 0.53 μs pixel dwell. Each pixel was excited 25 times in intervals of ~1 min each. After 45 min the brains were scanned again as indicated above.

### *In-vivo* imaging

To measure neuronal activity in the horizontal *Drosophila* mushroom body lobes, 3 to 6-day-old female flies expressing the respective sensors (GCaMP3.0, G-GECO1.1, G-GECO1.2, R-GECO1.0, or Synapto-pHluorin) were used. Flies were briefly anaesthetized on ice, immobilized in a small chamber with adhesive tape. A hole was cut through the head capsule for direct optical access. Tracheae were carefully removed and 1.5% low-melting agarose was injected into the head capsule to reduce the movement of the brain. The preparation was covered with Ringer's solution (Estes et al., 1996) and optical imaging was performed using a two-photon microscope (LSM7 MP, Zeiss) equipped with mode-locked Ti-sapphire Chameleon Vision II laser (Coherent) tuned to 690–1064 nm, a 500–550 m band-pass filter for green fluorescent sensors and a 575–610 nm band-pass filter for R-GECO1.0, and a Zeiss w-plan Apochromat 20 × water immersion objective (NA = 1.0). Images were acquired at a frame rate of 5 Hz with an excitation wavelength of 920 and 950 nm for green or red fluorescent sensors, respectively. Odor stimuli (4-methylcyclohexanol and 3-octanol, diluted in mineral oil 1:750 and 1:500, respectively) were applied in an air stream to the flies' antennae for 2 s each using a custom-built olfactometer at an air flow rate of 1 l/min. Three stimulations with each odor were applied to each individual fly with an interstimulus interval of 20 s. Images were acquired using the Zeiss ZEN software and images were later aligned in the X-Y direction using a MatLab program to correct for slight movements of the preparation (Guizar-Sicairos et al., 2008). Changes in fluorescence emission were calculated within a region of interest covering the horizontal lobes as ΔF/F_0_ where *F* is the fluorescence measured at each time point and *F*_0_ the baseline fluorescence before odor stimulation. *F*_0_ is calculated as the average of 5 frames before odor onset. For each fly the ΔF/F_0_ values of the 3 stimulations were averaged. To illustrate the spatial distribution of odor-evoked Ca^2+^ increases, false-color coded images were created. Three frames of baseline fluorescence directly preceding the odor onset were averaged and then subtracted from the average of 3 frames (400–1000 ms after stimulus onset) covering the peak of the fluorescence increase.

## Results

### Expression of fluorescence sensor proteins in kenyon cells using the duplicated mb247 promoter

**Figure 1:**
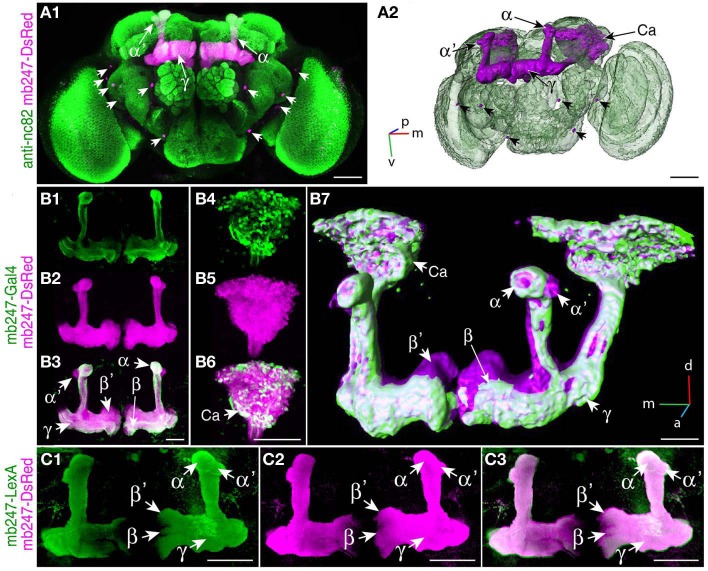
**Targeting the *Drosophila* mushroom body using a duplicated mb247 promoter construct. (A)** Expression of DsRed in the mushroom bodies of a *Drosophila* brain (frontal view, **A1**). Expression in neurons outside the mushroom body is indicated by arrowheads. Neuropils are stained using an anti-bruchpilot-antibody (green) and the DsRed expression is shown in magenta. **(A2)** 3D reconstruction of the brain depicted in **(A1)**. **(B)** Expression of mcd8-GFP under control of mb247-Gal4 is mainly confined to the α/β- and γ-lobes **(B1)**. **(B2)** On the contrary, DsRed expression under control of the duplicated mb247 promoter is visible also in the α′/β′-lobes. **(B3)** Overlay of GFP expression shown in **(B1)** and DsRed expression shown in **(B2)**. **(B4–B6)** Mushroom body calyx showing expression of GFP driven by mb247-Gal4 **(B4)**, expression of DsRed driven by the mb247 promoter construct **(B5)**, and an overlay of both **(B6)**. **(B7)** 3D reconstruction of the brain depicted in **(B1–B6)**. **(C)** GFP expression induced by mb247-LexA::VP16 (Pitman et al., 2011) is visible in α/β-, α′/β′-, and lobes γ-lobes **(C1)**. **(C2)** DsRed expression under control of the mb247 promoter. **(C3)** Overlay of **(C1)** and **(C2)**. Scale bars = 50 μm in **(A,B)**, and 100 μm in **(C)**. Ca, calyx; α, α-lobe; α′, α′-lobe; β, β-lobe; β′, β′-lobe; γ, γ-lobe; p, posterior; m, medial; v, ventral.

We used a duplicated DNA construct of the promoter mb247 (Schulz et al., 1996; Zars et al., 2000) to direct the expression of transgenes to the mushroom bodies (Table 1). This strategy has been described before by Riemensperger et al. (2005), who expressed the red fluorescence protein DsRed (Matz et al., 1999) in Kenyon cells. Pitman et al. (2011) created a LexA::VP16 driver line using the same mb247 promoter construct. Using these published fly lines, we first confirmed that the mb247 promoter-driven transgene expression encompasses all types of Kenyon cells, i.e., covers all lobes. Indeed, as can be seen by the fluorescence of DsRed (Figures 1A1,A2), all mushroom body lobes express the fluorescence protein. Transgene expression driven by the mb247 promoter is not completely restricted to the mushroom body and shows some “non-specific” expression. In addition to Kenyon cells, several scattered somata are visible in each hemisphere, located in the lateral suboesophageal ganglion and the protocerebrum (Figures 1A1,A2). Four to six cell somata are located directly below the γ-lobes of the mushroom bodies and form projections into the antennal lobes; 3–5 somata are located in the dorsal protocerebrum, and their projections can be traced to the ipsi- and contralateral anterior lobe region and the medial protocerebrum. Mb247-promoter-driven DNA constructs are, therefore, not exclusively but very predominantly expressed in Kenyon cells. In contrast, expression directed to the mushroom body by the conventional mb247-Gal4 driver line (Zars et al., 2000) does not label α′/β′-lobes, and the other lobes are not entirely labeled either (Figures 1B1–B7). The cores of the α/β-lobes are, for example, less densely labeled (Figure 1B). The mb247-LexA::VP16 driver line described by Pitman et al. (2011), on the contrary, induces an expression pattern in Kenyon cells that completely overlaps with the expression of mb247-DsRed (Riemensperger et al., 2005) (Figures 1C1–C3), which confirms that the more encompassing expression pattern induced by the duplicated mb247 promoter construct is reproducible across transgenic fly lines.

**Table 1:** **Summary of fly strains expressing transgenes directly under control of the duplicated mb247-promoter**.

**Fly strain**	**Application**	**References of fly strain**	**References of transgene**
mb247-DsRed	Anatomical landmark	Riemensperger et al., 2005	Matz et al., 1999
mb247-LexA	Driver for lexA/lexAop binary expression system	Pitman et al., 2011	Lai and Lee, 2006
mb247-Gal80	Repression of Gal4 expression	Krashes et al., 2007	Lee and Luo, 1999
mb247-GCaMP3.0	Ca^2+^-imaging	This study	Tian et al., 2009
mb247-G-GECO1.1	Ca^2+^-imaging	This study	Zhao et al., 2011
mb247-G-GECO1.2	Ca^2+^-imaging	This study	Zhao et al., 2011
mb247-R-GECO1.0	Ca^2+^-imaging	This study	Zhao et al., 2011
mb247-Synapto-pHluorin	pH-dependent imaging of neurotransmission	This study	Miesenböck et al., 1998
mb247-splitGFP11	Anatomical indicator of cell-cell contacts	Pech et al., 2013	Feinberg et al., 2008
mb247-C3paGFP	Back-tracing of individual cells	This study	Ruta et al., 2010
mb247-flippase	Intersectional/mosaic targeting of cells	This study	Bohm et al., 2010

**Figure 2:**
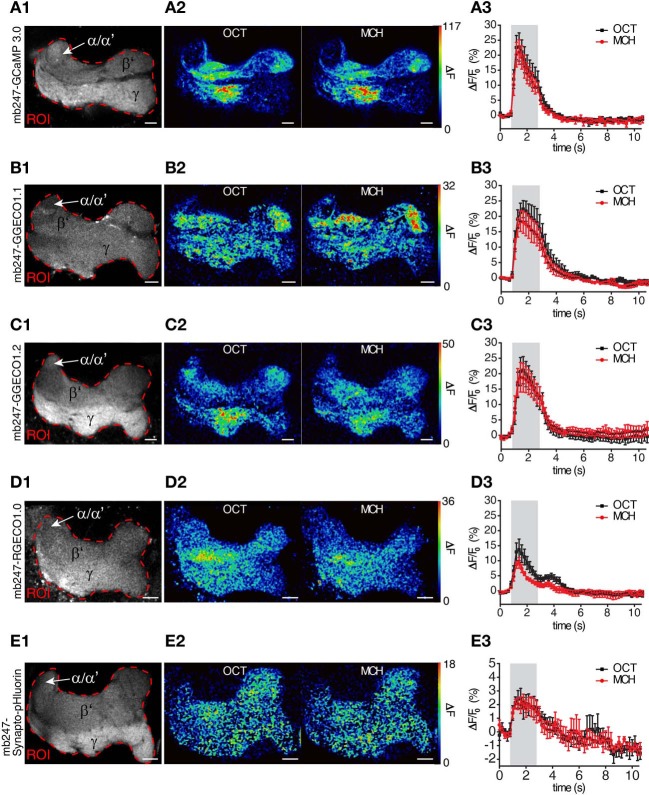
**Mb247-driven expression of fluorescent sensor proteins.** Optical imaging of odor-evoked neuronal activity in Kenyon cells of the adult *Drosophila* mushroom body using different reporter proteins, i.e., **(A1–A3)** the Ca^2+^ sensor GCaMP3.0, **(B1–B3)** the Ca^2+^ sensor G-GECO1.1, **(C1–C3)** the Ca^2+^ sensor G-GECO1.2, **(D1–D3)** Ca^2+^ sensor R-GECO1.0, and **(E1–E3)** Synapto-pHluorin. The left row **(A1–E1)** illustrates the fluorescence protein expression in one focal plane covering the horizontal lobes of the mushroom body. The dashed red line indicates the region of interest (ROI) in which odor-evoked changes in fluorescence emission were monitored. The middle row **(A2–E2)** shows two false-color coded images illustrating the spatial distribution of fluorescence intensity changes with the ROI evoked by 3-octanol (OCT) and 4-mehylcyclohexanol (MCH). The right row **(A3–E3)** shows the temporal dynamics of relative changes in fluorescence within the ROI evoked by OCT (black line) and MCH (red line). The odor stimulus is indicated as gray bars. Relative changes in fluorescence are indicated as means ± SEM (*n* = 5 animals each). α/α′, part of the vertical α/α′-lobes; β′, β′-lobe; γ, γ-lobes. Scale bars = 10 μm.

We used the duplicated mb247 promoter to drive the expression of fluorescent Ca^2+^ sensor proteins. The intracellular Ca^2+^ level closely correlates with neuronal excitation (Berridge, 1998; Burgoyne, 2007). Optical Ca^2+^ imaging represents, therefore, a widely used technique to monitor the activity of neurons in general (Grienberger and Konnerth, 2012), and also in the central brain of *Drosophila* (Riemensperger et al., 2012). We first created transgenic flies expressing the widely-used fluorescence Ca^2+^ sensors GCaMP3.0 (Tian et al., 2009) under control of the mb247 promoter and performed two-photon optical Ca^2+^ imaging experiments with the focus on the horizontal lobes. Although the overall expression pattern induced by the promoter construct is recapitulated with this transgene as well, baseline fluorescence was more pronounced in the γ-lobes when compared to the β′-lobes (Figure 2A1), which might reflect a higher tissue density or, alternatively, higher intracellular baseline Ca^2+^ levels. When the flies were stimulated with the odorants 3-octanol or 4-methylcyclohexanol, which are volatile chemicals that are often used for olfactory learning experiments in *Drosophila*, clear increases in intracellular Ca^2+^ were observed (Figures 2A1–A3). Relative fluorescence changes (ΔF/F_0_) averaged across the entire horizontal lobes reached 23.9 ± 4.4% for 3-octanol and 21.8 ± 4.3% for 4-methylcyclohexanol (mean ± sem, *n* = 5 each) (Figure 2A3). The time course of intracellular Ca^2+^ dynamics is characterized by a rapid increase in fluorescence emission starting with stimulus onset, a slight, adaptive decay during stimulation and a fast decay after stimulus offset. Recently, a novel subfamily of GCaMP-type Ca^2+^ sensor proteins has been engineered and named GECOs (Zhao et al., 2011). This development raised our attention because a red fluorescent version, R-GECO1.0, has been invented that can be combined with green fluorescent sensor or marker proteins (e.g., Tewson et al., 2012; Li et al., 2013). In addition, several green variants (G-GECOs) with different dissociation constants (*K*_*d*_) for Ca^2+^ have been created. We have created flies expressing G-GECO1.1 and G-GECO1.2 that differ in their Ca^2+^ affinities with *K*_*d*_ values of 0.62 μM Ca^2+^ and 1.15 μM Ca^2+^ (Zhao et al., 2011) under control of the mb247 promoter. Flies expressing these sensors show detectable baseline fluorescence in Kenyon cells (Figures 2B1,C1), which we noticed to be clearly lower when compared to the baseline fluorescence of GCaMP3.0. Just as with GCaMP3.0, intracellular Ca^2+^ influx evoked by the two odors is detectable in the horizontal lobe regions of the mushroom bodies as a spatially distributed pattern (Figures 2A2,B2,C2). Relative maximum changes in G-GECO1.1 fluorescence (ΔF/F_0_) evoked by the two odorants are similar to GCaMP3.0 with 22.7 ± 3.0% for 3-octanol and 18.7 ± 3.3% for 4-methylcyclohexanol (mean ± sem, *n* = 5 each). The kinetics of signal on- and offset are also comparable with GCaMP3.0 (Figure 2B3). Likewise, the G-GECO1.2 version shows equivalent changes in fluorescence emission intensity of 21.3 ± 4.5% for 3-octanol and 19.9 ± 4.5% for 4-methylcyclohexanol (mean ± sem, *n* = 5 each) (Figure 2C3). Drastic differences between the three types of sensor proteins were not observed under the experimental conditions used here, except for lower baseline fluorescence in the G-GECO type sensors. The red fluorescent version, R-GECO1.0 (Zhao et al., 2011), however, has the advantage that its emission wavelength is complementary to the green emission of many other transgenic tools relying on GFP variants. R-GECO1.0 is clearly expressed under control of the mb247 promoter (Figure 2D1). Baseline fluorescence is, however, under the conditions used here (two-photon excitation) drastically lower than that of GCaMP3.0. Relative changes in fluorescence elicited by the two odorants is also much smaller with 14.2 ± 3.4% evoked by 3-octanol and 9.7 ± 1.6% evoked by 4-methylcyclohexanol (mean ± SEM, *n* = 5 each) (Figures 2D2,D3). It must be noted that the lower baseline fluorescence and Ca^2+^-dependent increase in emission intensity might result from the two-photon excitation which is not optimized for exciting this red fluorescence protein. In fact, regular single-photon excitation at green light wavelengths causes stronger emission intensities. However, and also under our experimental conditions, the red fluorescence sensor is clearly functional in intrinsic mushroom body neurons. The time course of the odor-evoked change in fluorescence differs in the red R-GECO1.0 when compared to the green sensors. The ΔF/F_0_ signal decays back to baseline already during odor stimulation and shows a second peak following odor offset. This time course might more accurately reflect the sparse on- and offset action potential firing that has been described for Kenyon cells, e.g., using electrophysiological recordings in moths (Ito et al., 2008) or Ca^2+^ imaging with Fura-2 in honey bees (Szyszka et al., 2005).

Optical imaging of synaptic transmitter release using pH-sensitive GFP variants targeted to the lumen of synaptic vesicles represents a further method used to monitor the effect of neuronal activity. We have expressed Synapto-pHluorin (Miesenböck et al., 1998) under direct control of the mb247 promoter (Figure 2E1). Odor-evoked increases in fluorescence elicited by odors are detectable in the mushroom body lobes, with relative changes in fluorescence of up to 3.2 ± 0.7% evoked by 3-octanol and 2.9 ± 0.6% evoked by 4-methylcyclohexanol (Figures 2E2,E3). The ΔF/F_0_ peak is followed by a slow decay below baseline (~2%) due to bleaching of the fluorophore. The signal-to-noise ratio is, under these experimental conditions, drastically lower than that of Ca^2+^ imaging, which results from the physical constraints of the physiological parameter that is measured here. The fly strain is, however, clearly functional in reporting synaptic vesicle release, and if this parameter needs to be recorded in subregions of the mushroom body, this fly strain might provide a helpful tool.

### Mushroom-body directed expression of splitGFP

**Figure 3:**
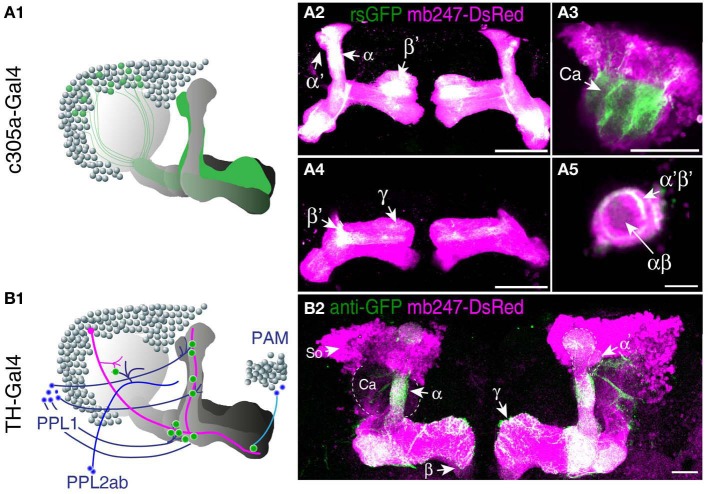
**Mushroom body-directed expression of reconstituted splitGFP to visualize cell–cell contacts within and between Kenyon cells. (A)** The fly strain mb247-DsRed; mb247-splitGFP11, UAS-splitGFP1-10 is crossed with c305a-Gal4. The offspring shows reconstituted splitGFP fluorescence (rsGFP) in Kenyon cells forming α′/β′- and γ-lobes, in addition to the DsRed fluorescence. **(A1)** Schematic illustration of the splitGFP reconstitution between populations of intrinsic mushroom body neurons determined by the c305a-Gal4 line and the mb247-promoter line. **(A2–A5)** Immunhistochemical visualization of the splitGFP reconstitution in the brain (frontal view) at different optical sections, i.e., at the level of the α/β- and α′/β′-lobes **(A2)**, the Calyx **(A3)**, the β′- and γ-lobes **(A4)**, and the peduncle **(A5)**. DsRed fluorescence is shown in magenta, reconstituted GFP fluorescence in green. **(B)** The fly strain mb247-DsRed; mb247-splitGFP11, UAS-splitGFP1-10 is crossed with TH-Gal4. The offspring shows reconstituted splitGFP fluorescence at contact regions of close proximity between populations of dopaminergic neurons and Kenyon cells, in addition to the DsRed fluorescence. **(B1)** Schematic illustration of the splitGFP reconstitution between populations of intrinsic mushroom body neurons and dopaminergic neurons from the PAM cluster, PPL1 cluster, and PPL2ab cluster of dopaminergic neurons. **(B2)** Reconstituted splitGFP fluorescence between dopaminergic neurons and Kenyon cells indicates contacts of dopaminergic neurons predominantly in the α- and γ-lobes and the heel of the horizontal lobes. Mb247-DsRed fluorescence is shown in magenta and the reconstituted splitGFP labeled by anti-GFP in green. Ca, calyx; So, somata; Scale bars = 10 μm in **(A5)**, 40 μm elsewhere.

A prerequisite for understanding the function of the mushroom body circuitry is detailed knowledge of the contacts between mushroom body intrinsic neurons (Kenyon cells) and mushroom body extrinsic neurons. The recently described GRASP technique (Feinberg et al., 2008) that has been adapted to *Drosophila* by Gordon and Scott (2009), provides an attractive tool to visualize and pinpoint where exactly two cells contact each other in close proximity and might potentially form synapses. We have recently reported a transgenic fly that expresses one part of splitGFP targeted to the outer surface of the cell membrane under control of the mb247 promoter (Pech et al., 2013). In addition, the mb247-DsRed construct is expressed as a landmark. The second part of the splitGFP is expressed under UAS control. If a given transgenic Gal4 strain is crossed with this “MB-splitGFP” fly strain, reconstitution between the two membrane-bound splitGFP parts can be visualized (Pech et al., 2013). Again, we would like to underline that, in contrast to the widely used mb247-Gal4 line (Zars et al., 2000), the mb247 promoter constructs cover all types of Kenyon cells including α′/β′-lobes (Riemensperger et al., 2005; Pech et al., 2013). First, the technique can be used to visualize Kenyon cells determined by a given Gal4 line. If both parts of the splitGFP are expressed in the same population of Kenyon cells, fluorescence is readily visible (Pech et al., 2013). This we exemplify here with the *Drosophila* line c305a-Gal4. This fly strain has been described as expressing Gal4 in the α′/β′-lobes (Krashes et al., 2007), and in fact, the α′/β′-lobes are labeled when c305a-Gal4 is crossed with MB-splitGFP (Figures 3A1–A5). In addition, faint splitGFP reconstitution is also observed in the γ-lobes (Figure 3A4). Secondly, the MB-splitGFP fly strain can also be used to visualize regions of close proximity between Kenyon cells and mushroom body extrinsic neurons (Pech et al., 2013). As an example, we have used the TH-Gal4 line (Friggi-Grelin et al., 2003), which covers a large proportion of tyrosine hydroxylase-immunoreactive neurons, i.e., several clusters of dopaminergic neurons in the *Drosophila* brain, in particular PPL1, PPL2ab, PPL2c, PAL, PPM1/2, and PPM3 clusters (Friggi-Grelin et al., 2003; Mao and Davis, 2009; Pech et al., 2013) (Figure 3B1). Reconstitution of a splitGFP signal at the contact points between these dopaminergic neurons and Kenyon cells is clearly visible in the α- and γ-lobes and the heel of the horizontal lobes, as already described in Pech et al. (2013) (Figure 3B2).

### Mushroom body-specific expression of photoactivatable GFP

**Figure 4:**
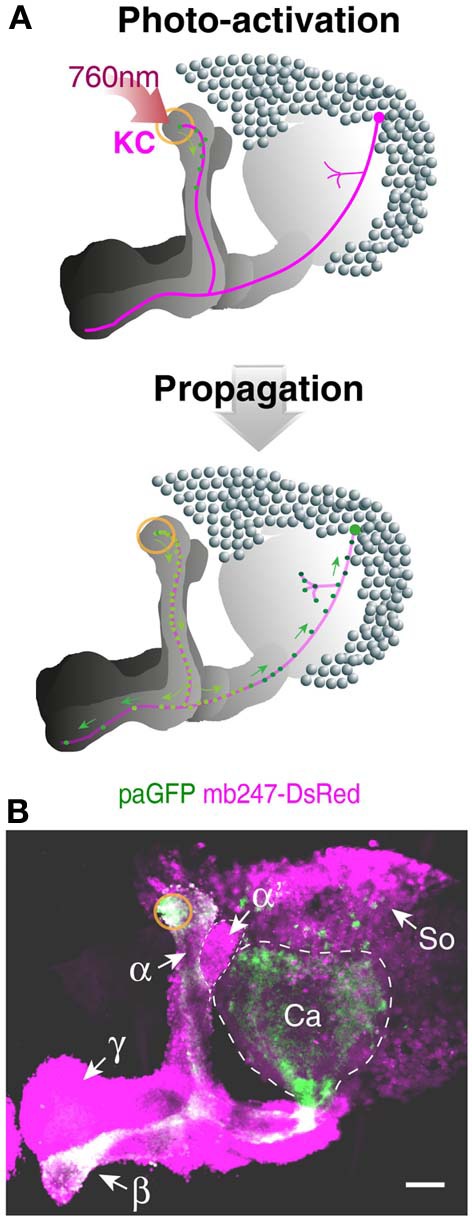
**Tracing Kenyon cells using photoactivatable GFP. (A)** Schematic depiction of photoactivated GFP (paGFP) signal (green) in defined populations of Kenyon cells in a frontal projection view. Mushroom body-localized paGFP is activated using a two-photon microscope at 760 nm in a region at the most posterior tip of the vertical α-lobe (upper panel). The photactivated paGFP molecules diffuse over time along the neurites and toward the cell bodies of the respective Kenyon cells (lower panel). **(B)** Fluorescence of paGFP in α-lobe neurons. The region of the paGFP activation is indicated by an orange circle. α, α-lobe; α′, α′-lobe; β, β-lobe; γ, γ-lobe; Ca, calyx; So, somata; KC, Kenyon cell. Scale bars = 40 μm.

The technique of photoactivating variants of GFP (Patterson and Lippincott-Schwartz, 2002) has been recently established in *Drosophila* to trace the neurites and projections of neurons from a particular point (Ruta et al., 2010; Pech et al., 2013). GFP-fluorescence is induced upon activation at 760 nm, and the photoactivated GFP diffuses along the neurites (Figure 4A). A paGFP expression allows, therefore, the visualization of isolated neurons of interest against a background of dense populations of neurons. We used the mb247 promoter sequence to express the variant C3paGFP (Ruta et al., 2010) globally in Kenyon cells and activated paGFP in a small, defined region at the most posterior tips of the vertical α-lobes (Figure 4A). The activated paGFP diffuses over time toward distal parts of the neurons and the cell bodies of the activated Kenyon cells. The paGFP signal can be traced from the activated region to the Kenyon cell somata at the level of the calyces and the tips of the posterior horizontal β-lobes (Figure 4B). If particular Kenyon cells need to be determined, e.g., by specific contacts with mushroom body extrinsic neurons, this fly strain might be helpful.

### Intersectional expression of transgenes in the mushroom body using mb247-flippase

**Figure 5:**
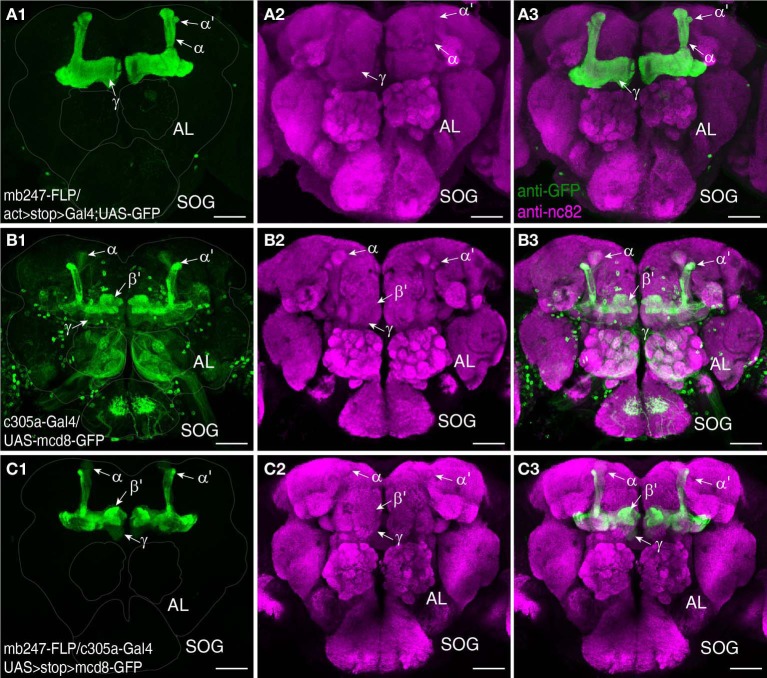
**Restriction of transgene expression to Kenyon cells using mb247-flippase. (A)** As an indicator for flippase activity a fly strain is used that carries a ubiquitous Act-Gal4 driver, with a FRT-flanked stop cassette preceding the Gal4 driver sequence. The mb247 promoter-induced flippase expression mediates the excision of the FRT-flanked stop cassette and causes transcription of Gal4 exclusively in the mushroom bodies. GFP expression is visualized using an anti-GFP antibody (green) **(A1)**, neuropils are visualized using an anti-bruchpilot antibody (magenta) **(A2)**. **(A3)** shows the overlay of **(A1,A2)**. **(B)** Expression pattern of c305a-Gal4 visualized by UAS:mcd8-GFP expression labeled by anti-GFP (green) **(B1)**. Neuropils are visualized using an anti-bruchpilot antibody (magenta) **(B2)**. **(B3)** shows the overlay of **(B1,B2)**. Flies show strong expression of GFP in the α′/β′ lobes and slight expression in the γ-mushroom body lobes and further strong expression outside the mushroom bodies predominantly in glomeruli of the antennal lobes (AL) and the subesophagial ganglion (SOG). **(C)** Flippase-mediated restriction of GFP expression labeled by an anti-GFP antibody (green) in c305a-Gal4 positive cells when UAS-FRT-stop-FRT-GFP is used. Flies show strong expression of GFP in the α/β- and slight expression in the γ-lobes, but no further expression outside the mushroom bodies **(C1)**. Neuropils are visualized using an anti-bruchpilot antibody (magenta) **(C2)**. **(C3)** shows the overlay of **(C1,C2)**. Scale bars = 50 μm.

Many Gal4-driver lines that are potentially interesting for the investigation of mushroom bodies show not only expression in Kenyon cells, but also exhibit non-specific Gal4 expression in other neurons of the brain or thoracic ganglia (Aso et al., 2009). In order to specify and restrict expression to the mushroom bodies we have made use of an intersectional approach based on the yeast flippase recombinase (Golic and Lindquist, 1989; Struhl and Basler, 1993; Xu and Rubin, 1993; Bohm et al., 2010). We generated a fly line that permanently expresses the flippase protein in the Kenyon cells of the mushroom body, which can be combined with a DNA insertion carrying flippase recognition target sites (FRT). The constitutive flippase activity in Kenyon cells will induce the removal of any FRT-flanked DNA sequences. We first demonstrate the spatial specificity of the flippase activity by crossing mb247-FLP with a line that carries an actin-FRT-stop-FRT-Gal4 sequence along with a UAS-GFP reporter (Pignoni and Zipursky, 1997). In the absence of flippase activity Gal4 expression, and therefore GFP expression, are prevented due to the preceding stop codon. Since the actin promoter drives expression ubiquitously in the brain, GFP expression reports, in this case, all cells in the brain that exhibit flippase activity; i.e., in the entire mushroom body (Figures 5A1–A3). This fly strain can be useful in multiple combinations. First, flippase activity in the mushroom body can be used to clip a stop codon that prevents transcription of a target gene, and thereby restrict gene expression to Kenyon cells. The intersectional logic of this “and” system is apparent when UAS-controlled transgenes are used that are preceded by a FRT-flanked stop cassette. UAS-induced expression is determined by a Gal4 driver pattern, but the transgenes are expressed only in these neurons that overlap with Kenyon cells. We show this by using the mb247-FLP line to restrict the expression pattern of a non-specific Gal4 line, c305a-Gal4 (Krashes et al., 2007) that drives Gal4 expression in a large number of neurons, e.g., in the antennal lobes or the suboesophageal ganglion of the *Drosophila* brain, in the mushroom body α′/β′-lobes, and, albeit faintly, in the γ-lobes (Figures 5B1–B3). When a UAS-FRT-stop-FRT-GFP reporter is used (Yu et al., 2010) mb247-FLP restricts the expression of the transgene efficiently to Kenyon cells. Flies now show a strong expression of GFP in the α′/β′-lobes and slight expression in the γ-lobes of the mushroom body, but no additional expression outside the mushroom bodies (Figures 5C1–C3). Of course, other applications of the mb247-FLP line are conceivable, e. g., removing a FRT-flanked Gal80 construct (Bohm et al., 2010) in order to restrict gene expression to all neurons except Kenyon cells, or to induce mitotic recombination during development (MARCM; Dang and Perrimon, 1992; Lee and Luo, 1999) in the mushroom body.

## Discussion

Discovering how brain circuits process and compute information and contribute to organizing behavior represents a key topic in current neuroscience. “Model animals” that can be genetically manipulated through the expression of transgenes, e.g., mice, zebrafish, *C. elegans*, or *Drosophila melanogaster*, are particularly favorable for this task. The mushroom body of the *Drosophila* brain provides a relatively delineated structure that can serve as a model circuitry to address fundamental aspects of neuronal processing in general. First, the mushroom body provides the possibility of analyzing how odor information is encoded and processed in central brain structures (Fiala, 2007; Masse et al., 2009). Fruit flies perceive odors with olfactory sensory neurons located on the third antennal segments and the maxillary palps (Vosshall and Stocker, 2007). These sensory neurons project to the antennal lobes, the structural and functional analogue of the olfactory bulb of vertebrates. Each olfactory sensory neuron expresses one or very few olfactory receptors, and those sensory neurons that express the same receptors target the same glomeruli in the antennal lobe (Vosshall and Stocker, 2007). As a consequence, odors are represented at the level of the antennal lobe in terms of overlapping, combinatorial activity of glomeruli (Fiala et al., 2002; Wang et al., 2003). Olfactory projection neurons transfer olfactory information to the lateral horn and the mushroom body where they form large boutons (Tanaka et al., 2004). The mushroom body's Kenyon cells contact these boutons and receive input from many projection neurons (Caron et al., 2013). Odors are represented at the level of the mushroom body in terms of highly selective, sparse activity of very few (~5%) Kenyon cells (Turner et al., 2008; Luo et al., 2010; Honegger et al., 2011). How the transformation from an overlapping, combinatorial odor code to a selective, sparse code is achieved, e.g., through the particular projection neuron to Kenyon cell connectivity, physiological properties of the respective neurons and inhibitory feedback loops, is subject to intense current research (Perez-Orive et al., 2002; Jortner et al., 2007; Turner et al., 2008; Luo et al., 2010; Honegger et al., 2011; Caron et al., 2013). The principle of converting spatio-temporal “codes” that reflect stimulus properties into “sparse codes” that do not directly reflect aspects of the original stimulus seems to be conserved across such evolutionarily distant species as mammals and insects (Leinwand and Chalasanim, 2011). Due to its relatively small number of neurons in comparison with cortical areas of the vertebrate brain, the mushroom body provides a favorable test system to analyze these topics. Second, the mushroom body also provides a favorable test system to investigate how experience-dependent changes in behavior caused by associative learning are mediated (Heisenberg, 2003; Davis, 2005; Fiala, 2007). Flies can be trained to associate an odor with a punishment or a reward (reviewed in Fiala, 2007), and synapses at the mushroom body lobes are believed to be a critical place for the coincidence of the two stimuli (Heisenberg, 2003). Particular dopaminergic neurons have been shown to mediate the punishment information during the associative learning (Schroll et al., 2006; Aso et al., 2010), whereas a different group of dopaminergic neurons mediates reward information (Liu et al., 2012). Octopaminergic neurons play an additional, modulatory role in reward learning (Hammer, 1993; Hammer and Menzel, 1998; Schroll et al., 2006; Burke et al., 2012). Investigating these two aspects of neuronal functioning requires appropriate experimental approaches.

Among all insects *Drosophila melanogaster* represents a favorable test organism because it is genetically manipulable and sophisticated genetic tools and expression systems have been invented to investigate the neuronal mechanisms underlying its behavior (Olsen and Wilson, 2008; Venken et al., 2011). These genetic techniques to dissect the structure and function of neuronal circuits encompass, first, anatomical methods to characterize the structure and connectivity of the constituting elements of the circuit, i.e., neurons and synapses. Second, genetic tools to monitor parameters of neuronal function have been engineered, e.g., Ca^2+^ sensors or fluorescence probes for synaptic vesicle release. Third, proteins to disrupt neuronal function or synaptic transmission can be expressed. And fourth, optogenetic and thermogenetic tools to artificially induce neuronal activity are available (Fiala et al., 2010; Riemensperger and Fiala, 2013). For analyzing specific parts of the mushroom body circuitry it is desirable to express combinations of transgenes in different populations of neurons. However, combinatorial expression of several transgenes is limited by the particular expression systems. One can combine the three binary expression systems that are available, i.e., the Gal4-UAS system (Brand and Perrimon, 1993), the LexA/LexAop system (Lai and Lee, 2006), and the Q system (Potter and Luo, 2011). Each expression system requires two genomic insertions, and the practical limitations in combining these transgenes are obvious. Mb247-driven expression of transgenes can be combined with one or two of the above-mentioned binary expression systems relatively simply (e.g., see Pech et al., 2013). The compilation of stable mushroom body-expressing transgenic *Drosophila* strains provided here might be of help in this regard.

### Targeting and visualizing the mushroom body

A visual landmark of the mushroom body helps to determine the spatial configuration of neurons relative to the mushroom body. The mb247-DsRed fly strain described by Riemensperger et al. (2005) has been used a number of times for that purpose (e.g., Lin et al., 2007; Tanaka et al., 2008; Claridge-Chang et al., 2009; Pech et al., 2013). As a prerequisite for physiological studies the visual landmark should ideally be detectable both *in vivo* and, for a *post-hoc* analysis, in fixed tissue, which is the case with DsRed. Here we describe two additional tools based on mb247-dependent transgene expression. Recently, Pech et al. (2013) adapted the GRASP technique (Feinberg et al., 2008; Gordon and Scott, 2009) and designed transgenic flies to visualize cell-to-cell contacts between Kenyon cells and mushroom body extrinsic neurons. It is often difficult to unambiguously identify and characterize putative innervations of potential mushroom body extrinsic neurons based on the expression of cytosolic or membrane-bound fluorescence proteins. The strategy of Pech et al. (2013) was to express one part of the membrane-bound split-GFP in the mushroom body and, in addition, a second part in a different subset of cells under UAS-control. This allows one to selectively visualize close proximity between intrinsic and extrinsic neurons. This tool can be combined with the expression of paGFP (Patterson and Lippincott-Schwartz, 2002; Ruta et al., 2010) under control of the mb247 promoter, which provides the possibility of tracking the anatomy of Kenyon cells from one particular Kenyon cell/extrinsic neuron contact up to the somata and axonal arborizations in different lobes. The combination of the mb247-DsRed, the mb247-splitGFP, and/or the mb247-paGFP in combination with high resolution microscopy, e.g., two-photon microscopy (Denk et al., 1990), might be helpful for detailed anatomical studies in the intact *Drosophila* brain. Of course, these anatomical markers can also be combined with genetically encoded fluorescence sensors as reporters of neural activity. A third approach relies on the expression of the yeast-derived flippase, which can be used to restrict the expression of marker or effector genes to Kenyon cells with relatively high specificity. The repertoire of, among others, reporter and effector genes coupled to FRT-flanked stop cassettes is constantly growing. This will allow for a restricted visualization or manipulation of mushroom body intrinsic cells included in the often very non-specific enhancer trap lines.

### Monitoring neuronal activity in the mushroom body

Since the very first description of genetically encoded Ca^2+^ sensors (Miyawaki et al., 1997; Romoser et al., 1997) and their first applications in *Drosophila* (Reiff et al., 2002; Fiala et al., 2002) continuous progress in their development has led to very improved versions of Ca^2+^ sensors. In particular, the invention of G-CaMP-type sensors (Nakai et al., 2001) has laid the foundation for engineering today's state-of-the art sensors (Tian et al., 2009; Zhao et al., 2011; Akerboom et al., 2012, 2013; Ohkura et al., 2012; Chen et al., 2013). Ratiometric FRET-based sensors, e.g., cameleon-type (Miyawaki et al., 1997) or troponin-based sensors like TN-XXL (Mank et al., 2008) are useful for particular applications. However, these sensors that are based on the simultaneous detection of YFP and CFP emission are difficult to be combined with the simultaneous detection of another wavelength. Therefore, we have chosen and directed four different single-wavelength Ca^2+^ reporters (GCaMP3.0, Tian et al., 2009; G-GECO1.1 and 1.2 and R-GECO1.0, Zhao et al., 2011) specifically to Kenyon cells under mb247 control. For a detailed and quantitative comparison of the four different Ca^2+^ reporters used, please refer to Walker et al. (2013) and Yamada and Mikoshiba (2012) The performance of the G-GECO1.1 and G-GECO1.2 (Zhao et al., 2011) has been reported to be comparable to that of the GFP-based GCaMP 3.0, with varying dynamic ranges and kinetics, however. Our results on odor-evoked Ca^2+^ dynamics in Kenyon cells are in accordance with these reports, and all three green fluorescent sensors expressed in the mushroom body are functional and reliably detect odor responses of Kenyon cells. However, the functionality of R-GECO1.0 (Zhao et al., 2011) has been discussed controversially (Yamada and Mikoshiba, 2012). When expressed in the mushroom body, R-GECO1.0 shows similar kinetics at signal onset as the two G-GECO indicators. However, it shows much lower maximal signal intensity. This lower efficiency of the red fluorescent sensor in comparison with the green ones might be simply due to a lower excitability using two-photon excitation in the infrared range. That might also explain the critical evaluation of R-GECO1.0 by Yamada and Mikoshiba (2012), who also used two-photon microscopy. An alternative red fluorescent Ca^2+^ sensor protein (RCaMP) has been described recently (Akerboom et al., 2013), which, on the one hand, appears favorable for two-photon excitation and simultaneous optogenetic activation of neurons using channelrhodopsin-2 (Akerboom et al., 2013). On the other hand, in a direct comparison (Akerboom et al., 2013) R-GECO1.0 shows higher sensitivity to detect action potentials, better signal to noise ratio and larger maximal fluorescence increase (ΔF/F_0_)_max_. We could confirm the high sensitivity of R-GECO1.0, as this was the only sensor that reported distinguishable on- and offset signals, mimicking the electrophysiological odor response of Kenyon cells (Ito et al., 2008). Further, R-GECO1.0 has been used successfully in the olfactory system of the *Drosophila* brain in combination with the green sensor GCaMP3.0 (Li et al., 2013). The development of novel Ca^2+^ sensors progresses constantly, and improved GCaMP variants are published very frequently (Tian et al., 2009; Zhao et al., 2011; Akerboom et al., 2012, 2013; Chen et al., 2013). The pCaSpeR-mb247 vector is available if one wishes to create additional fly strains that express novel sensors under mb247 promoter control.

However, Ca^2+^ imaging is not the only method of observing the activity of neurons. Sometimes it is advantageous to monitor synaptic transmitter release, in particular in the context of functional interactions between Kenyon cells and mushroom body extrinsic neurons. Therefore, we targeted Synapto-pHluorin (Miesenböck et al., 1998) to Kenyon cells. Compared to the Ca^2+^ sensors, Synapto-pHluorin shows a relatively small fluorescence increase in response to the odor onset. The low signal-to-noise ratio as can be estimated by the relation between its low signal and the relatively large standard errors. Only an estimated fraction of up to 5% of synaptic vesicles are used for release and reuptake at an active synapse (Denker et al., 2011), and Synapto-pHluorin is also expressed in the remaining 95% of vesicles of the reserve pool. The poor signal-to-noise ratio is, therefore, a result of the parameter measured.

In *Drosophila*, there is a fast-growing and impressive library of multipartite expression systems and their variants and modifications (Duffy, 2002; Pfeiffer et al., 2008, 2010; Bellen et al., 2011; Venken et al., 2011) to target more and more neuronal subsets or single neurons with greater precision. This study aims at complementing these genetic techniques for the specific application of analyzing a particular neuronal circuit, the mushroom body. If transgenes are expressed under direct control of the mb247 promoter the commonly used binary transcription systems are still available to express, in addition, marker proteins, fluorescence sensors, optogenetic, and thermogenetic actuator proteins. The broad palette of fly strains assembled here might be of help in this task.

## Author contributions

Ulrike Pech created and characterized the mb247-splitGFP strain, the mb247-paGFP strain, and the mb247-GCaMP3.0 strain, performed immunohistochemistry and two-photon microscopy. Shubham Dipt created the mb247-Synapto-pHluorin strain and performed all *in-vivo* optical imaging experiments. Jonas Barth created the mb247-GECO strains, Priyanka Singh created the mb247-FLP strain, Mandy Jauch tested the mb247-lexA strain, Andreas S. Thum provided the DNA of the original pCaSpeR-mb247-vector and created the mb247-Gal80 strain, André Fiala conceived the experiments, wrote the manuscript together with TR and Thomas Riemensperger created the mb247-DsRed strain, performed immunohistochemistry, analyzed data, supervised experiments and wrote the manuscript together with AF.

### Conflict of interest statement

The authors declare that the research was conducted in the absence of any commercial or financial relationships that could be construed as a potential conflict of interest.
